# Prevalence and associated factors of dysmenorrhea among secondary and preparatory school students in Debremarkos town, North-West Ethiopia

**DOI:** 10.1186/s12905-018-0552-x

**Published:** 2018-04-24

**Authors:** Abebaw Abeje Muluneh, Tewodros seyuom Nigussie, Kahsay Zenebe Gebreslasie, Kiber Temesgen Anteneh, Zemenu Yohannes Kassa

**Affiliations:** 1 Department of Midwifery, College of Medicine and Health Sciences, Hawassa University, PO Box- 1560, Hawassa, Ethiopia; 2 Department of Midwifery, College of Medicine and Health Sciences, University of Gondar, PO Box-196, Gondar, Ethiopia; 3 Department of Midwifery, College of Health Sciences, Mekelle University, PO Box- 1871, Mekelle, Ethiopia

## Abstract

**Background:**

Dysmenorrhea is one of the most common gynecologic disorders and a frequently observed cause of anxiety and discomfort among female adolescents. Its prevalence varies between 16% and 91% in women of reproductive age. Its population statistics are too scant in Ethiopia. This study was aimed to determine the prevalence and associated factors of dysmenorrhea among secondary and preparatory school students in Debremarkos town, 2016.

**Methods:**

Institutional based cross-sectional study was employed from Sept.26 to Oct.17, 2016 among secondary and preparatory school students in Debremarkos town. Self-administered questionnaire was used to collect data from 539 individuals selected by simple random sampling technique. Data were checked, coded and entered into Epi-data version 3.1 and exported to SPSS version 20 for analysis. Univariate, bivariate and multivariable analysis were carried out. Binary logistic regression model was computed and *P* value < 0.05 was considered as significant. All ethical procedures were considered.

**Results:**

The prevalence of dysmenorrhea was 69.3%. Age, AOR (95% CI) =1.38(1.15, 1.65), family history of dysmenorrhea, AOR (95% CI) = 9.79(4.99, 19.20), physical activity, AOR (95% CI) =0.39(0.13, 0.82), sugar intake, AOR (95% CI) =2.94 (1.54, 5.61), early menarche AOR (95% CI) =4.10(1.21,13.09), late menarche AOR (95% CI) =0.50 (0.27, 0.91), heavy menstrual periods AOR (95% CI) =2.91(1.59, 5.35) and sexual intercourse AOR (95% CI) =0.24 (0.10.0.55) had statistically significant association with the occurrence of dysmenorrhea.

**Conclusions:**

Age, positive family history of dysmenorrhea, physical activity, excessive sugar intake, early menarche, late menarche, sexual intercourse and heavy menstrual periods had a statistically significant association with the occurrence of dysmenorrhea.

## Background

Dysmenorrhea is a pain associated with menstruation which can be abdominal pain, backache, stomach cramp, waist pain and/or thigh pain [1–4]. It can be primary, in the absence of an identifiable organic pathology, or secondary, with demonstrable pathology [5–7]. Primary dysmenorrhea usually started at or shortly after (6 to 12 months) menarche, when ovulatory cycles are established [7, 8]. The pain usually starts along with menstrual bleeding and lasts from 48 to 72 h [9].

Dysmenorrhea is the most commonly reported menstrual disorder [10] and one of the most common gynaecologic disorders [11, 12]. It affects a large proportion of women of reproductive age [10]. It is usually sever enough to result in a significant socioeconomic dysfunction and disability particularly in adolescents and young women. It has a major impact on women’s quality of life, work productivity, and health-care utilization [13]. It is the leading cause of morbidity leading to limitation of daily activities and recurrent school absenteeism among adolescents [6, 14]. Generally, it has a negative impact on social, academic, and sport activities of many female adolescents [5, 15]. Hence, failure to address dysmenorrhea causes a major social and financial burden for families, neighbors, communities and the glob at large.

Understanding the full range of dysmenorrhea among adolescent girls is a crucial point in developing appropriate management and preventive strategies. But studies on the status of dysmenorrhea and associated factors are scarce in Ethiopia. Hence, this study was aimed to determine the prevalence of dysmenorrhea and identify associated factors among secondary and preparatory school students in Debremarkos town.

## Methods

### Study design and period

A cross sectional study was conducted from September 26 to October 17, 2016 in Debremarkos town.

### Study area

Debremarkos town is 300Km Northwest of Addis Ababa, the capital of Ethiopia, and 265Km Southeast from Bahir Dar, the Regional capital city of Amhara national regional state. Based on the 2007 population and housing census, the total population size of the town was estimated to be 62,469 of which 47.9% were male and 52.1% were females. From this, 14,618 were women in the reproductive age group [16]. Currently, there are five, four governmental and one private, secondary and preparatory schools in the town serving for a total of 7483 students of which 3564 are females.

### Sampling

For this study, sample size was determined using single population proportion formula by taking the proportion of dysmenorrhea 72% in Dabat and Kolla diba [17] at 95% confidence interval (CI) and an acceptable difference of 4%.

Considering 10% none response rate, the total sample size was 539.

There are five secondary and preparatory schools in the study area. Female students from grades 9 to 12 were included in the study. After having the list of all female students with their respective grades and sections from each school, codes were assigned for each student, and a new sampling frame was constructed. Simple random sampling technique was used. By using these numerical codes, study subjects were drown by OpenEpi, computer software.

### Operational definitions

**Dysmenorrhea**: for this study, an adolescent was considered to have dysmenorrhea if she had one or more of abdominal pain, groin/pelvic pain, back pain or thigh pain before and/or during her menstrual periods for the last one year.

**Physical activity:** the habits of physical activity was measured by the participants’ self-report of not at all, irregularly or regularly.

**Daily allowance:** participants’ daily allowance was considered adequate or inadequate depending on their subjective report.

**Non-academic duty:** participants who didn’t involve in any duty at home or outside were classified in “not at all” group; those participating in activities like home cleaning, coffee making, bed making, washing their own clothes, were grouped in “simple category”; whereas, participants involved in activities such as daily labor, marketing activities, cooking activities, farming activities were considered as “heavy”.

**Sugar intake:** Excessive if individuals took 12 or more teaspoons of table sugar daily, Moderate if 6 to 12 teaspoons; and in a restricted use if less than 6 teaspoons [18].

**Menstrual characteristics:** For the purpose of this study, menstrual patterns were characterized as follows:

Long cycle: cycle repeated once every > 35 days but < 3 months, short (frequent) cycle: cycle repeated once every < 21 days, short duration: duration of menses < 3 days, long duration: duration of period > 7 days, and Heavy cycles were considered if a lady changes 5 or more sanitary/ vulvar pads per day but scant if one or less.

### Data collection

Data were collected using a self-administered questionnaire designed in English and translated to Amharic and back to English for consistency. It has three parts, the first containing socio-demographic information of the study participants. The second part address the life style and behavioral issues including physical activity, sugar intake, coffee intake, tea intake as well as alcohol drinking, smoking and chat chewing habits. The final part was asking about reproductive issues and menstrual pattern.

The questionnaire was pre-tested on 27 (5% of the total sample) school girls in Finoteselam town and necessary modifications were made. A one day training on the overall steps and procedures of data collection and proper data handling was provided for the data collectors and supervisor.

The data were collected by 4 female diploma teachers and supervised by one high school teacher. Voluntary assent was obtained from each participant. The questionnaire was distributed to every participant and collected on the same day to ensure confidentiality and prevent information contamination.

Throughout the course of the data collection, regular meetings were held among the data collectors, supervisor and the principal investigator. Two more additional visits were made for participants who were not available during the first visit. The collected data were reviewed and checked for completeness before data entry and the incomplete data were discarded. Data entry template was prepared.

### Data analysis

Data were checked, coded and entered to Epi-data version 3.1 and exported to SPSS (Statistical Package for Social science) version 20 for analysis. Data entry was made by the principal investigator. Texts, tables and graphs were used to present the result.

Binary logistic regression model was computed to test the presence of association between dependent and independent variables. First, bivariate analysis was computed for each predictor variable. Then, all predictor variables with *P* < 0.2 were included in the multivariable analysis. Finally, significance was considered at *p* < 0.05 with 95% CI.

## Results

In this survey, a total of 539 individuals were included with a response rate of 94.8%. Thirteen participants were not voluntary to participate and 15 questionnaires were found to be incomplete and excluded from the analysis.

### Sociodemographic characteristics of the respondents

The study was conducted among 443 (86.7%) urban and 68 (13.3%) rural female secondary and preparatory school students. Their age was in the range of 14 to 24 years with the mean age of 17.55 ± 1.62 years. From the total respondents, 458 (89.6%) were in the age range of 15-19 years. Five hundred four (98.6%) of the study participants were Amhara in ethnicity and 489 (95.7%) were followers of Orthodox Christianity. Only 18 (3.5%) were ever married. The mean family size in which the participants have been living was 5.24 ± 2.13 ranging from 1 to 14 (Table 1).

**Table 1:** Socio demographic characteristics of secondary and preparatory female students in Debremarkos town, North West Ethiopia, 2016

Variables		frequency (*n* = 511)	Percent (%)
Age	10-14	6	1.2
	15-19	458	89.6
	20-24	47	9.2
Religion	Orthodox	489	95.7
	Protestant	16	3.1
	Muslim	6	1.2
	^a^Others	7	1.4
Mothers’ educational status	Can’t read and write	137	26.8
	Can read and write	140	27.4
	Primary school	64	12.5
	Secondary school	75	14.7
	College and above	95	18.6
Participants living with	With her parents	406	79.5
	With her husband	10	2.0
	Alone in a rent house	84	16.4
	With others	11	2.2
Family size	≤4	193	37.8
	5-8	279	54.6
	≥9	39	7.6
Daily allowance (*n* = 492)	Adequate	295	60.0
	Inadequate	197	40.0
Family history of dysmenorrhea	Yes	203	39.7
	No	237	46.4
	I don’t know	71	13.9

^a^Others = Catholic, Johba

### Life style and behavioural characteristics of the respondents

More than half (54.2%) of the respondents didn’t involve in any physical activity. Three hundred fifty seven (70%) of the students involved in simple non-academic duties in the family. None of the respondents had smoked and used chat (Table 2).

**Table 2:** Life style and behavioral characteristics of secondary and preparatory female students in Debremarkos town, North West Ethiopia, 2016

Variables		Frequency (*n* = 511)	Percent (%)
Physical activity	Not at all	277	54.2
	irregularly	175	34.2
	Regularly	59	11.5
Non-academic duty in the family	not at all	89	17.4
	Simple	358	70.1
	Heavy	64	12.5
Daily coffee intake in cups	0	248	48.5
	1-4	194	38.0
	≥5	69	13.5
Tea intake	Yes	489	95.7
	No	22	4.3
Drinking alcohol	not at all	456	89.2
	Irregularly with occasions	36	7.0
	regularly	19	3.7
Sugar intake	Excessive	169	33.1
	Moderate	330	64.6
	Minimal	12	2.3
Chronic illness	Yes	62	12.1
	No	449	87.9
Chronic or recurrent pelvic pain	Yes	17	3.3
	No	494	96.7

### Reproductive characteristics and menstrual patterns of the participants

The mean age at menarche was 13.16 ± 1.76 years with a range of 9-17 years. About half (49.7%) of the respondents experienced menarche in the age range of 13-14 yeas. Thirty nine (7.6%) individuals had history of sexual intercourse of which 19 (48.7%) had history of contraceptive use. Only 1 (2.6%) had pregnancy history. Three hundred eighty three (75%) of the total respondents had regular menstrual cycles, of which 360 (94.0%) had normal menstrual cycle length (21-35 days). The menstrual cycle length ranges from 14 to 40 days with the mean cycle length of 28.29 ± 2.81 days. The duration of menstrual flow was from 1 to 11 days with the mean of 4.41 ± 1.60 days. Four hundred fourteen (81%) individuals had normal duration of menstrual flow (3-6 days) (Table 3).

**Table 3:** Reproductive characteristics and menstrual patterns of secondary and preparatory female students in Debremarkos town, North West Ethiopia, 2016

Variables		Frequency (*n* = 511)	Percent (%)
Age at menarche	9-12	115	22.5
	13-14	254	49.7
	15-17	142	27.8
Types of contraceptive used (*n* = 20)	OCPs	9	45.0
	injectable	6	30.0
	implants	5	25.0
Menstrual cycle pattern	Regular	383	75.0
	Irregular	128	25.0
Menstrual cycle length in days (*n* = 383)	< 21	16	4.2
	21-35	360	94.0
	> 35	7	1.8
Duration menstrual flow in days	1-2	18	3.5
	3-6	414	81.0
	≥7	79	15.5
Amount of menstrual flow	heavy	181	35.4
	Normal	292	57.1
	Light	38	7.4

### Patterns of dysmenorrhea among the participants

Of the total 511 respondents, 354 (69.3%) had dysmenorrhea. For 168 (47.5%) and 144 (40.7%) of the respondents with dysmenorrhea, the pain started 1-2 days before and just after the onset of their menses respectively. Two hundred seventy two (76.8%) experienced dysmenorrhea during every menstrual periods (Table 4).

**Table 4:** Patterns of dysmenorrhea among secondary and preparatory school students in Debremarkos town, North West Ethiopia, 2016

Patterns of dysmenorrhea		frequency (*n* = 354)	Percent (%)
Beginning/ starting of the pain	1-2 days before the onset of menses	168	47.5
	just after the onset of menses	144	40.7
	a day after the onset of menses	33	9.3
	two days after the onset of menses	4	1.1
	3 days before the onset of menses	5	1.4
Occurrence of the pain	in every period	272	76.8
	one or two periods apart	47	13.3
	only when periods are during exam or hard working times	35	9.9
Time when the pain relieved after the onset of menses	< 1 day	29	8.2
	1-2 days	142	40.1
	3-5 days	167	47.2
	> 5 days	16	4.5
Total duration of the pain	< 1 day	41	11.6
	1-2 days	111	31.4
	3-5 days	193	54.5
	> 5 days	9	2.5

### Factors associated with dysmenorrhea

On multivariable analysis, increasing age, AOR (CI) =1.38(1.15, 1.65), positive family history of dysmenorrhea, AOR 95% (CI) = 9.79(4.99, 19.20), excessive sugar intake, AOR (CI) =2.94(1.54, 5.61), early menarche (≤12 years), AOR (95% CI) = 4.10(1.21, 13.09), late menarche (≥15 years), AOR (95% CI) = 0.50(0.27, 0.91), being sexually active, AOR (95% CI) = 0.24(0.10, 0.55) and heavy menstrual flow, AOR (95% CI) = 2.91(1.59, 5.35) had statistically significant association with the occurrence of dysmenorrhea(Table 5).

**Table 5:** Bivariate and multivariable analysis of factors associated with dysmenorrhea among secondary and preparatory school students in Debremarkos town, North West Ethiopia in 2016

Variables	Dysmenorrhea		Crude OR(95%CI)	AOR (95% CI)
	Yes (%)	No (%)		
Age (years)	354(69.3)	157(30.7	1.24(1.07, 1.45)	1.38(1.15,1.65)**
Nonacademic duty				
Not at all	55(61.8)	34(38.2)	1.00	1.00
Simple	245(68.6)	112(31.4	3.33(1.50,7.42)	1.63(0.61,4.36)
Heavy	54(84.4)	10(15.6)	1.35(0.84,2.19)	1.23(0.66,2.28)
Family history of dysmenorrhea				
Yes	190(93.6)	13(6.4)	14.25(7.7,26.41)	9.79(4.99,19.20)**
No	120(50.6)	117(49.4)	1.00	1.00
I don’t know	44(62.0)	27(38.0)	1.59(0.92, 2.73)	1.98(0.89, 3.76)
Habit of physical activity				
not at all	216(78)	61(22)	1.00	1.00
irregularly	106(60.6)	69(39.4)	0.43(0.29,0.66)	0.57(0.34,0.94)*
regularly	32(54.2)	27(45.8)	0.34(0.19,0.60)	0.39(0.19,0.85)*
Daily coffee intake (cups)				
0	156(62.9)	92(37.1)	1.00	1.00
1-4	135(69.6)	59(30.4)	1.35(0.91, 2.01)	1.63(0.99,2.67)
≥5	63(91.3)	6(8.7)	6.12(2.58, 14.8)	2.13(0.47, 8.36)
Sugar intake				
Excessive	145(85.5	24(14.2)	3.88(2.39,6.30)	2.94(1.54,5.61)**
Moderate	201(60.9	129(39.1)	1.00	1.00
In a restricted amount	8(66.7)	4(33.3)	1.28(0.38,4.35)	1.57(0.52,4.73)
Age at menarche (years)				
≤12	109(94.8)	6(5.2)	10.27(4.3,24.29)	4.10(1.21,13.09)*
13-14	161(63.1)	91(36.1)	1.00	1.00
≥15	83(58.5)	59(41.5)	0.80(0.52,1.21)	0.50(0.27,0.91)*
History of intercourse				
Yes	21(53.8)	18(46.2)	0.49(0.25,0.94)	0.24(0.10,0.55)**
No	333(70.6)	139(29.4)	1.00	1.00
Duration of menstrual flow				
≤2	10(55.6)	8(44.4)	0.64(0.25,1.65)	0.48(0.16,1.38)
3-6	274(66.2)	140(33.8)	1.00	1.00
≥7	70(88.6)	9(11.4)	3.97(1.93,8.19)	1.91(0.85,4.27)
Amount of menstrual flow				
Heavy	158(87.3)	23(12.7)	4.72(2.88,7.76)	2.91(1.59,5.35)**
Normal	173(59.2)	119(40.8)	1.00	1.00
Light	23(60.5)	15(39.5)	1.06(0.53,2.12)	0.80(0.33,1.94)

*= > *P* < 0.05; **= > *P* < 0.001

## Discussion

The prevalence of dysmenorrhea in this study was found to be 69.3% with 95% CI of (65.2, 73.4%). This was in line with 66.8% in Debre Berhan University [2], 71.8% in Mekelle University [10] and 72% among secondary school adolescents in Dabat and Koladiba, northwest Ethiopia [19]. It was also comparable with 65.4, 69.8 and 72.7% reported among university students in Egypt [5], Nigeria [17] and Turkey [20, 21] respectively. However, it was higher than 64% among university students in Mexico [22], 45% among young college Nursing students in India [8] and 61.27% in Rural South Africa [23]. This difference may be explained by the sociocultural differences of the study groups in pain perception and threshold as well as the difference in life style. It was also less than that of in university of Gondar and Bahr Dar University, northwest Ethiopia [5, 24] which reported a prevalence rate of 77.6 and 85.1% respectively. This inconsistency is probably due to the fact that the prevalence of dysmenorrhea increases with age up to the pick age (24 years) where the age in Bahir Dar University ranges 17 to 24 with mean age of 20.4 ± 1.2 years but in this study it was 14-24 and 17.55 ± 1.62 years respectively. Moreover, this difference may be resulted from the absence of universally accepted or standardized method of defining dysmenorrhea or from its subjective measurement. Furthermore, this study revealed a lower prevalence of dysmenorrhea than 73.83% reported among university students in India [14] which may be because of the sociocultural differences of the study groups in pain perception and threshold, life style and age.

The prevalence of dysmenorrhea, in this study, increased with increasing age, AOR (95% CI) =1.38(1.15, 1.65). This is supported by studies conducted in Isfahan University in Iran [25], Lebanese [26] and among Mexican university students [22] which identified a statistically significant association between age and dysmenorrhea. Similarly, a highly significant difference between the ages of the women with and without dysmenorrhea was reported in Turkey [7]. However, no significant difference was observed between the mean age of women with and without dysmenorrhea in south India [27]. The inconsistency might be because of the difference in the age of the study participants. The age in this study was from 14 to 24 year, but it was from 11 to 28 years in south India which include both the age ranges below and above the pick age.

This study identified a statistically significant association between the occurrence of dysmenorrhea and positive family history of dysmenorrhea, AOR (95% CI) 9.79 (4.99, 19.20). This is consistent with the findings of similar studies in Serbia [1], Turkey [10, 20, 21], India [11], Lebanese [25], Mekelle [10] and Bahir Dar Universities in Ethiopia [24]. This is probably related to genetic factors, which needs further studies.

In this study, physical activity was found to have a statistically significant association with the occurrence of dysmenorrhea, AOR (95% CI) = 0.399 (0.19, 0.85), which was consistent with the findings in a systematic review of randomized controlled trials, which confirms as exercise reduces the symptoms associated with dysmenorrhea [28]. But, no statistically significant association was observed between physical activity and dysmenorrhea in Egypt [4]. The difference may be because of measurement difference; i.e. in this study physical activity was measured by self-report of the participants’ habit of physical activity as not at all, irregularly or regularly, where as in Zagazig University, Egypt participants were asked as if they were athletes, active in physical activity or sedentary.

In this study, a statistically significant association between excessive sugar intake and the presence of dysmenorrhea was observed, AOR (95% CI) =2.94(1.54, 5.61), which was similar with the result of a cross-sectional surveys in Turkey [20]. This is also supported by statistically significant association between dysmenorrhea and consumption of more than four glasses of tea per day among Debre Berhan University students in Ethiopia [12]. This is probably because food items reach in sugar may contain the precursors of prostaglandins which are presumed to be the cause of dysmenorrhea. This requires further investigations.

A statistically significant association between age at menarche and dysmenorrhea was identified in this study. This was supported by studies conducted among high school students in Mexico, and university students in Mexico, Serbia, Palestinian and Egypt [1, 2, 4, 22]. Early menarche was related to an increase in the prevalence of dysmenorrhea, AOR (95% CI) =4.10(1.21, 13.09). Whereas, late menarche was associated with the decrease in the prevalence of dysmenorrhea, AOR (95% CI) =0.50(0.27, 0.91). This is also supported by the thought that dysmenorrhea is caused by the release of inflammatory factors during menstruation [29] and started after the establishment of ovulatory menstrual cycles [30].

In this study, a statistically significant association between the presence of dysmenorrhea and heavy menstrual flow was observed, AOR (95% CI) = 2.91(1.59, 5.35), which was consistent with a report among Mexican high school students [2], Heavy menstrual flow was related to increased prevalence of dysmenorrhea which is also in line with the thought that dysmenorrhea is caused by the release of inflammatory factors during menstruation [29]. This is further strengthened by a more likelihood of having dysmenorrhea during long menstrual flow periods in Mekelle University [10].

### Limitations of the study

Readers, please note that since most variables in this study were measured by the participants’ subjective-report, observation and recall bias may be introduced.

## Conclusions

The prevalence of dysmenorrhea among secondary and preparatory school students in Debremarkos town was high.

Dysmenorrhea was more common with increasing age, among ladies with positive family history of dysmenorrhea, excessive sugar intake habit, early menarche and those having heavy menstrual periods. Whereas, physical activity, late menarche and sexual intercourse were found to be protective factors for the occurrence of dysmenorrhea.

## Abbreviations

AOR: Adjusted Odds Ratio
CI: Confidence Interval
OR: Odds Ratio

## References

[1] Adinma ED, Adinma JI (2008). Perceptions and practices on menstruation amongst Nigerian secondary school girls. Afr J Reprod Health.

[2] Ortiz MI, Rangel-Flores E, Carrillo-Alarcón LC, Veras-Godoy HA (2009). Prevalence and impact of primary dysmenorrhea among Mexican high school students. Int J Gynecol Obstet.

[3] Hillen TI, Grbavac SL, Johnston PJ, Straton JA, Keogh JM (1999). Primary dysmenorrhea in young western Australian women: prevalence, impact, and knowledge of treatment. J Adolesc Health.

[4] Nooh AM (2015). Menstrual disorders among Zagazig University students, Zagazig, Egypt. Middle East Fertil Soc J.

[5] French L (2005). Dysmenorrhea. Am Fam Physician.

[6] Davis AR, Westhoff CL (2001). Primary dysmenorrhea in adolescent girls and treatment with oral contraceptives. J Pediatr Adolesc Gynecol.

[7] Osayande AS, Mehulic S (2014). Diagnosis and initial management of dysmenorrhea. Am Fam Physician.

[8] Shah M, Monga A, Patel S, Shah M, Bakshi H (2013). A study of prevalence of primary dysmenorrhea in young students-a cross-sectional study. Indian J Community Med.

[9] Deligeoroglou E, Tsimaris P (2010). Menstrual disturbances in puberty. Best Pract Res Clin Obstet Gynaecol.

[10] Aktas D (2015). Prevalence and factors affecting dysmenorrhea in female university students: effect on general comfort level. Pain Manage Nurs.

[11] Omidvar S, Bakouei F, Amiri FN, Begum K (2015). Primary dysmenorrhea and menstrual symptoms in Indian female students: prevalence, impact and management. Global J Health Sci.

[12] Ylikorkala O, Dawood MY (1978). New concepts in dysmenorrhea. Am J Obstet Gynecol.

[13] Ju H, Jones M, Mishra G (2014). The prevalence and risk factors of dysmenorrhea. Epidemiol Rev.

[14] Singh A, Kiran D, Singh H, Nel B, Singh P, Tiwari P (2008). Prevalence and severity of dysmenorrhea: a problem related to menstruation, among first and second year female medical students. Indian J Physiol Pharmacol.

[15] Banikarim C, Chacko MR, Kelder SH. Prevalence and impact of dysmenorrhea on Hispanic female adolescents. Arch Pediatr Adolesc Med. 2000;154 10.1001/archpedi.154.12.1226 11115307

[16] Encyclopaedia of Debre-Markos town administration. Socioeconomic characteristics; 2011.

[17] Amaza DS, Sambo N, Zirahei JV, Dalori MB, Japhet H, Toyin H. Menstrual pattern among female medical students in University of Maiduguri, Nigeria. Br J Med Med Res. 2(3):327–37. 2012 Jul-Sep

[18] World Health Organization (2015). Guidlines: sugars intake for adults and children.

[19] Zegeye DT, Megabiaw B, Mulu A (2009). Age at menarche and the menstrual pattern of secondary school adolescents in Northwest Ethiopia. BMC Womens Health.

[20] Ozerdogan N, Sayiner D, Ayranci U, Unsal A, Giray S (2009). Prevalence and predictors of dysmenorrhea among students at a university in Turkey. Int J Gynecol Obstet.

[21] Unsal A, Ayranci U, Tozun M, Arslan G, Calik E (2010). Prevalence of dysmenorrhea and its effect on quality of life among a group of female university students. Ups J Med Sci.

[22] Ortiz MI (2010). Primary dysmenorrhea among Mexican university students: prevalence, impact and treatment. Eur J Obstet Gynecol Reprod Biol.

[23] Oni TH, Tshitangano TG (2015). Prevalence of menstrual disorders and its academic impact amongst Tshivenda speaking teenagers in rural South Africa. J Hum Ecol.

[24] Shiferaw MT, Wubshet M, Tegabu D. Menstrual problems and associated factors among students of Bahir Dar University, Amhara National Regional State, Ethiopia: a cross-sectional survey. Pan Afr Med J. 2014;17 10.11604/pamj.2014.17.246.2230 PMC4189866 25309646

[25] Habibi N, Huang MSL, Gan WY, Zulida R, Safavi SM (2015). Prevalence of primary dysmenorrhea and factors associated with its intensity among undergraduate students: a cross-sectional study. Pain Manage Nurs.

[26] Santina T, Wehbe N, Ziade F (2012). Exploring dysmenorrhoea and menstrual experiences among Lebanese female adolescents. Eastern Mediterr Health J.

[27] Omidvar S, Bakouei F, Amiri FN, Begum K (2016). Primary dysmenorrhea and menstrual symptoms in Indian female students: prevalence, impact and management. Glob J Health Sci.

[28] Brown J, Brown S. Exercise for dysmenorrhoea. Cochrane Database Syst Rev. 2010;(2):Cd004142. 10.1002/14651858.CD004142.pub2 20166071

[29] Balbi C, Musone R, Menditto A, Prisco LD, Cassese E, D'Ajello M (2000). Influence of menstrual factors and dietary habits on menstrual pain in adolescence age. Eur J Obstet Gynecol Reprod Biol.

[30] Harel Z (2006). Dysmenorrhea in adolescents and young adults: etiology and management. J Pediatr Adolesc Gynecol.

